# Herd Prevalence Estimation of *Mycobacterium avium* Subspecies *paratuberculosis* Burden in the Three Main Dairy Production Regions of Germany (PraeMAP)

**DOI:** 10.3390/ani12040447

**Published:** 2022-02-12

**Authors:** Susanne Eisenberg, Mette Krieger, Amely Campe, Ingrid Lorenz, Esra Einax, Karsten Donat

**Affiliations:** 1 Niedersächsische Tierseuchenkasse, Anstalt des öffentlichen Rechts, 30169 Hanover, Germany; 2 Department of Biometry, Epidemiology and Information Processing, WHO Collaborating Centre for Research and Training for Health at the Human-Animal-Environment Interface, University of Veterinary Medicine, 30559 Hanover, Germany; mette.krieger@tiho-hannover.de (M.K.); amely.campe@tiho-hannover.de (A.C.); 3 Bavarian Animal Health Service, 85586 Poing, Germany; ingrid.lorenz@tgd-bayern.de; 4 Thüringer Tierseuchenkasse, Anstalt des Öffentlichen Rechts, 07745 Jena, Germany; eeinax@thtsk.de (E.E.); kdonat@thtsk.de (K.D.); 5 Klinikum Veterinärmedizin, Klinik für Geburtshilfe, Gynäkologie und Andrologie der Groß- und Kleintiere mit Tierärztlicher Ambulanz, Justus-Liebig-Universität Giessen, 35390 Giessen, Germany

**Keywords:** paratuberculosis, cattle, MAP-control program, MAP prevalence

## Abstract

**Simple Summary:**

Paratuberculosis, caused by *Mycobacterium avium* ssp. *paratuberculosis* (MAP), is widely spread among ruminants worldwide. After a long-lasting incubation period, infected animals suffer from chronic granulomatous enteritis. Economic losses are caused by premature culling, reduced milk yield and slaughter value in the dairy and beef industry, triggering attempts to control the disease. Paratuberculosis is a listed disease (category E), according to the European Animal Health Law, and intended to be monitored within the European Union. Evaluation of several herd-level monitoring approaches including the testing of environmental fecal samples to detect the infectious agent have been evaluated, proving environmental sampling to be a useful monitoring tool on herd level. This study comprises the application of environmental sampling within a herd prevalence estimation study in German dairy herds. Based on regional differences in the structure of livestock farming, Germany was divided into three regions where a representative number of farms were visited for sample collection. The results clearly indicate a different regional MAP herd level prevalence. The highest percentage of affected herds is found in the eastern part with large dairy herds, and the lowest in the south with the smallest average herd size. We conclude that the regional differences in MAP prevalence imply different approaches to control the disease.

**Abstract:**

On-farm environmental sampling is an effective method for herd-level diagnosis of *Mycobacterium avium* ssp. *paratuberculosis* (MAP) infection and between-herd prevalence estimation. So far, no prevalence study enrolling important livestock-farming regions has been conducted. As the structure of dairy farming differs between main livestock-farming regions in Germany, our objective was to assess the between-herd prevalence of paratuberculosis for these regions in a standardized approach. Methods: In total, 457 randomly selected dairy farms from three regions of Germany (North: 183, East: 170, South: 104) were sampled between 2017 and 2019. Environmental samples (boot-swabs, aggregate feces and/or liquid manure samples) were cultured and analyzed using an IS900-qPCR for MAP determination. Of the 457 selected farms, 94 had at least one MAP-positive environmental sample with significant differences between regions regarding the apparent (North: 12.0%, East: 40.6%, South: 2.9%) or corrected true (North: 14.8%, East: 50.1%, South: 3.6%) between-herd prevalence. In conclusion, regional differences of between-herd prevalence of paratuberculosis are substantial in Germany, indicating the need for control approaches with different aims. Taking into account regional MAP prevalence, MAP-control programs should focus on on-farm prevalence reduction or on mitigating the risk of between-herd transmission, depending on region.

## 1. Introduction

Paratuberculosis, or Johne’s disease, is a bacterial infection of the small intestine caused by *Mycobacterium avium* ssp. *paratuberculosis* (MAP). MAP is an acid-fast rod-shaped organism, which can survive in the environment for several months [1]. The infection is mainly present in farmed cattle, sheep and goat around the world but is also found in wild living species such as deer. Fecal shedding of MAP occurs in infectious animals, whereas oral uptake of MAP from the environment by susceptible animals leads to transmission of the disease [2]. Due to the long incubation time and the slow disease progression, early identification of infected animals is difficult. Infected animals start shedding MAP intermittently before clinical symptoms are present [2].

On an animal level, paratuberculosis can be diagnosed using ELISA for antibody detection in milk and blood samples or by using PCR and culture methods to detect MAP DNA in fecal samples [2]. In addition, for MAP herd status determination, environmental sampling has been described as an easy, cheap and effective sampling strategy [3].

Paratuberculosis has been described as early as 1895 by Johne and Frothingham [4]. It has spread around the world with reported herd-level prevalence between 7% and 83%, depending on the production type of cattle herds studied and the diagnostic methods used [5,6]. In general, herd-level prevalence tends to be lower in beef herds compared with dairy herds and for dairy herds it has been found to be positively related to herd size [7,8]. The comparison of MAP-prevalence between studies should be interpreted with caution when determined by different diagnostic methods. So far, data to compare MAP-prevalence between German regions using the same test approach are currently missing. These knowledge gaps hamper prevention and control of paratuberculosis [9]. Nevertheless, based on either laboratory or non-laboratory data herd-level prevalence was estimated to be above 40% in dairy cattle in most developed countries. In addition, prevalence is believed to be underestimated in most countries mainly because of the type of tests used in combination with a lack of monitoring [8]. 

Paratuberculosis is a listed disease according to the European Animal Health Law (category E), and intended to be monitored within the European Union. Diagnostic methods for monitoring are not defined yet (Regulation (EU) No. 2018/1882). In Germany, a passive monitoring system is in place where MAP-positive results have to be reported to the authorities under certain conditions. However, so far, there is no active, monitoring program to facilitate regular animal testing, and a prevalence study using a standardized approach is missing. Therefore, available prevalence estimates for federal German states can only be compared with caution. Estimates for apparent herd-level prevalence data based on different study designs and test methods exist for a few federal states, varying from 8.9% in Hessia up to 30.2% in Thuringia and Saxony [7,10,11,12]. The available data suggest regional differences in MAP prevalence but due to different study designs and especially because of different test approaches, numbers should be interpreted cautiously. Nonetheless, marked regional differences of livestock farming throughout Germany [13] indicate that different regional between-herd MAP prevalences can be expected. 

The objective of this study was to determine between-herd MAP prevalence in the three main livestock-farming regions of Germany. Participating farms were identified using a stratified sampling procedure. 

## 2. Materials and Methods

### 2.1. Herd Selection

**Figure 1:**
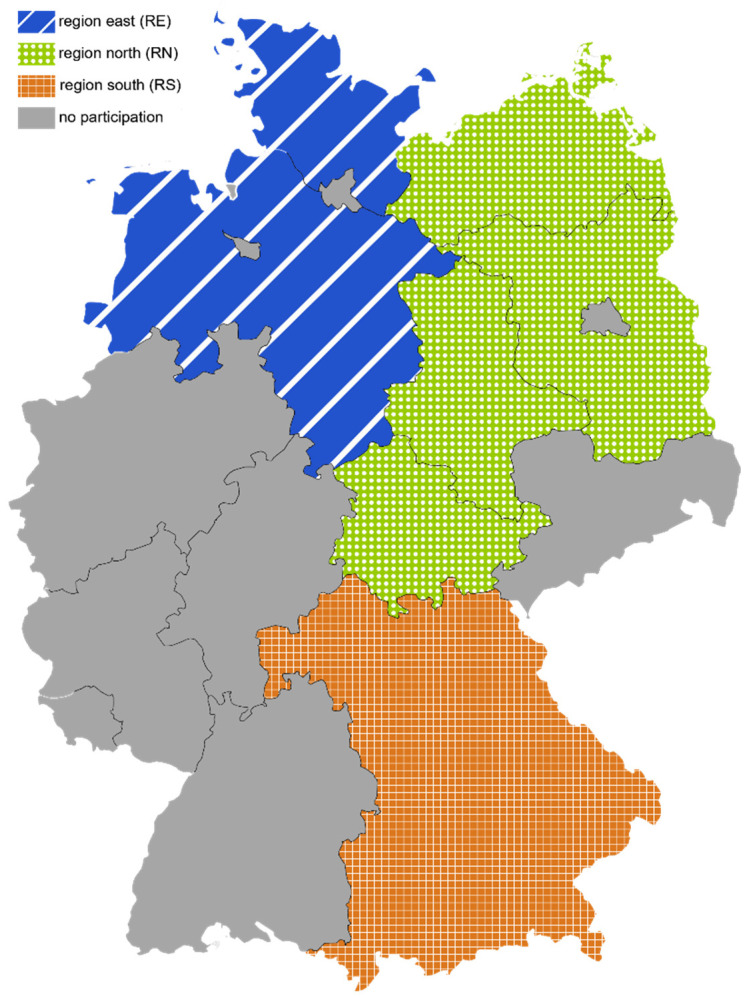
Federal states of Germany and the 3 regions defined for this study.

Within the framework of a joint project (PraeRi study) of the University of Veterinary Medicine Hannover Foundation, the Faculty of Veterinary Medicine of Freie Universität Berlin and the Faculty of Veterinary Medicine of Ludwig-Maximilian-Universität Munich dairy farms were visited, and animal health, biosecurity and animal housing were scored [14]. Participating farms were located either in Schleswig-Holstein and Lower Saxony (region north [RN]), in Brandenburg, Mecklenburg-Western Pomerania, Thuringia, Saxony-Anhalt (region east [RE]) or in Bavaria (region south [RS]) (Figure 1). Enrolled farms were randomly selected for the PraeRi study from the German database on animal identification and registration (HI-Tier) [14,15] and stratified according to herd size. Participating farmers of the PraeRi project were invited to participate in the MAP prevalence study (PraeMAP) by written consent. Participation in both studies was voluntary. Three research teams based in either Hanover, Berlin or Munich performed farm visits. In RN and RE environmental samples for this study were collected during the initial farm visit. In RS sampling on participating farms was performed by veterinarians of the Bavarian Animal Health Service during a separate farm visit. 

Between July 2017 and July 2019, the research teams contacted farmers and conducted the on-farm visitation and sampling. Due to an a priori training in environmental sampling, further permanent training and the usage of standard operation procedures (SOP), observer and sampler effects were considered negligible during the study.

### 2.2. Sampling Methods

Environmental sampling was performed using boot-swabs, slurry and environmental fecal sampling [11,16,17], as laid down in a written SOP. In short, boot-swabs were collected by walking between 100 and 200 steps in high cow traffic areas, e.g., waiting pen and main cow alleyway as described before [16]. After walking, the boot-swabs were placed in an aseptic plastic bag and labelled. Slurry was mixed before a sampling container was submerged in the liquid storage area to collect a sample of 150 mL slurry. Environmental fecal samples were collected by sampling 5 to 10 locations in high cow traffic areas. Boot-swab and slurry samples were the preferred way of sampling. Whenever a slurry sample was not available, an environmental fecal sample was collected. 

After collection, samples were stored at −20 °C with a maximum of three months at the respective university until transportation to and analysis at the laboratory of the Thuringian Animal Health Service in Jena (RN and RE) or at the Bavarian Animal Health Service in Poing (RS).

### 2.3. Laboratory Analysis

Samples were submitted to fecal culture for detection of viable MAP as well as to IS900 real-time PCR or detection of direct MAP DNA. Before analysis, boot-swabs were washed in NaCl to extract fecal material as described before [17]. Supernatants of the boot-swabs, slurry and environmental samples were analyzed by fecal culture according to the official guidelines of diagnostic procedures published by the Friedrich-Loeffler-Institut (FLI), the German Federal Research Institute for Animal Health [18,19]. Characteristic colonies were heated and sonicated according to a standardized method recommended by the FLI for DNA isolation [18] and confirmed by a conventional IS900 PCR [20]. Only samples with typical grey-white colonies with mycobactin-dependent growth, and a positive IS900 PCR were characterized as MAP-culture-positive [18]. 

In addition, a commercial IS900 real-time PCR was applied on each fecal sample for direct detection of MAP without a preceding cultivation step. For direct DNA extraction (QIAmp DNA Mini kit, Qiagen, Hilden, Germany) and sample pre-concentration (Adifil 100, Adiagene, Bio-X Diagnostics S.A., Rochefort, Belgium) for MAP DNA detection commercial kits were used according to the manual provided by the manufacturer. For MAP DNA detection a commercial IS900-based real-time PCR method was used (Adiavet Paratb, Adiagene, Belgium) according to the manufacturer’s instructions. 

Farms were categorized as MAP-positive when at least one sample had either a MAP-positive culture or direct PCR outcome or both.

### 2.4. Data Analysis

Data analysis was performed using the statistical software package SAS [21] and EPITOOLS [22]. Farms were categorized as MAP-positive if at least one of the collected samples tested MAP-positive. Apparent between-herd prevalence (AP) for each region was calculated. Herd-level sensitivity of the MAP-detection method was determined by applying fecal culture and/or the Adiavet Paratb qPCR test on a set of a boot swab and a slurry sample derived from a data set of a previous study [23] as follows: fecal culture—0.72%, real-time—PCR 0.78%, both tests combined—0.81%, and herd-level specificity of fecal culture was 1.00. The specificity of the Adiavet Paratb PCR test was determined using the validation data given in the manual of the manufacturer (1.00). True prevalence estimates (TP) [24] and 95% confidence intervals for AP and TP [25] were calculated for each region. In order to account for sporadic false positive results that may be due to pre- or postanalytical sample handling in practical application, we performed a sensitivity analysis for the calculation of the TP using the specificity estimates of 0.99 and 0.98.

## 3. Results

### 3.1. Study Farms

A total of 457 (RN = 183; RE = 170; RS = 104) farmers agreed to participate in the study. Farm characteristics differed per region (Table 1). Median herd size between regions differed substantially with the largest farms in RE and the smallest farms in RS. Holstein Friesians represented the typical breed in farms located in RN and RE, whereas in RS, Simmenthal was the main breed. The percentage of cattle housed in free stalls was highest in RN compared with RE and lowest in RS. Results regarding animal health in general, biosecurity and animal housing will be published elsewhere.

### 3.2. Boot-Swabs and Environmental Samples 

In total, results of 367 environmental samples were available of RN, 341 of RE and 205 of RS. An overview regarding the detection of MAP DNA and viable MAP per kind of environmental sample is given in Table 2. As expected, for most samples, both detection methods showed positive results (column ‘both’) and only for a minority of samples’ PCR and culture results were not congruent (column ‘PCR pos’ and ‘culture pos’).

### 3.3. Between-Herd Prevalence 

Apparent MAP between-herd prevalence differed markedly between regions (Table 3). In the eastern part of Germany 40.59% [Confidence Interval (CI) 33.21; 47.97] of herds were detected MAP-positive whereas in the southern region only 2.88% [CI 0; 6.09] were MAP-positive. The true apparent prevalence was only slightly higher compared with the AP (Table 3). 

However, the assumption that test specificity in field samples is lower (0.98) than 1 led again to minor changes in TP. The corrected between-herd TP of RE decreased to 48.9 [39.5; 58.2] and in RS to 1.1 [0; 5.2].

## 4. Discussion

This study aimed at estimating the prevalence of MAP-positive dairy herds in three main livestock-farming German regions using an environmental sampling protocol as an easy-to-use and cost-effective sampling approach [26]. Both fecal culture and a MAP IS900 real-time PCR were used to detect the presence of viable MAP and/or MAP DNA, as previously reported [19,23]. The need of this study resulted from missing a prevalence study regarding paratuberculosis at national level where farms were selected using a stratified selection process and the same protocol for sampling and analysis. The three chosen regions relate to the three main livestock farming regions (North, East, South) as determined by Merle et al. [13], which represent the structural diversity of the German dairy industry. Sharing the sampling frame of the joint project PraeRi enabled us to perform a study with uniform stratified random farm selection ensuring representativeness for each region. 

As expected, a clear north–south divide of MAP between-herd prevalence was detected, with more affected farms in the northern part of Germany than in the southern part, and an excessively high percentage of MAP-positive herds was identified in the eastern region. These regional differences of MAP prevalence were expected because of the regional diversity of farming [13]. In addition, existing regional studies with convenient herd selection and sampling as well as different diagnostic methods had already pointed in that direction. However, comparison of those data had to be performed cautiously due to the different approaches. For example, by testing pooled milk for MAP-specific antibodies within the mandatory MAP-control program of Lower Saxony an apparent prevalence of 20% was calculated [12], which is slightly higher than the point estimate of TP determined here, but lies within the 95% CI. A Bavarian study reported a higher herd-level seroprevalence, namely 12% (95% CI: 5–15%), using an ELISA test with a specificity of 99% compared with 3.56% between-herd prevalence established here, presumably due to sampling bias of the convenience sampling in the former study [27]. Based on our results, we assume that the differences between our results and results reported by the former study may be caused by the limited specificity of the used tests and the missing calculation of TP. Another reason for overestimating prevalence might have been convenience sampling with an overrepresentation of affected herds, because those herds may be more willing to test their herd and therefore introducing bias. This underlines the value of the uniform stratified random farm selection performed in our study. Two studies based on serological testing were published for regions in the eastern part of Germany reporting an apparent between herd-level prevalence of 85% or 90% [28,29]. These findings were also biased by low specificity of the applied ELISA test with 77% or 95%, respectively. Furthermore, these studies used data from voluntary control programs in the respective regions. As mentioned above, the affected herds are more likely to be enrolled in voluntary control programs. Both effects may have led to an overestimation of herd-level prevalence in these studies. A study [7] based on environmental sampling (boot swab) and applying a commercial real-time PCR reported 30% MAP-positive herds resulting in herd-level TP estimates of 56% for the federal state Thuringia and 34% for Saxony. Nonetheless, the results presented here are in line with those of previous studies in Germany and confirm the suitability of environmental sampling to estimate the herd-level prevalence of paratuberculosis. Using the same diagnostic approach, prevalence studies at national level were performed in the United States of America (USA) and Canada as well, substantiating a true between-herd prevalence of 91% for USA and 24–66% for dairy herds in different Canadian regions [30,31]. 

Herd size has been identified to be positively related to the risk of a MAP-positive herd-status [7,10,31,32]. This is assumed to be a relevant factor for the difference between the regions in our study, where the southern region with the lowest mean herd size had the lowest herd-level prevalence, and MAP was most present in herds of the eastern region with a median herd size of 199 cattle. This effect may be explained by the higher propensity of large herds to buy cattle, or a denser occupation of the calving pen may play a role, which is also linked to within-herd transmission of MAP [33,34]. Furthermore, keeping dairy cows in free stalls is positively associated with a positive MAP status when compared with tie stalls [10,31], which again is more common in small herds in southern Germany compared with large herds in the eastern part. 

Based on the fact that we used the same sampling scheme and MAP detection method in all three regions, we assume a good internal validity of our study. Between-sampler variability was minimized using written SOP protocols for collecting slurry and environmental samples and for the use of boot swabs. Evaluating inter-observer reliability was not considered because the additional herd-level data used in this study to describe the different structure of the dairy industry in the respective regions (herd size, breed, housing) was easy to record. Furthermore, categorizing these traits in dichotomous variables (German Holstein breed or not, free stalls or not) reduced the risk of misclassification. Due to sample management samples collected in this study were stored at −20 °C up to three months before analysis. It has been discussed that MAP viability might be reduced by longtime freezer storage and repeated freezing and thawing cycles. Since we limited storage time to a maximum of three months and samples were only thawed once before analysis the influence on MAP survival can be neglected [35]. With respect to the laboratory methods, a high level of quality assurance is established in both laboratories that performed the fecal culture and the real-time PCR test. Both laboratories are accredited veterinary test laboratories under license of the German Accreditation Body according to the quality standards of the German and European Standard DIN EN ISO 17025. The standard includes ring trails and comparative test between laboratories. The same commercial real-time PCR test system was applied in both laboratories, and fecal culture was performed according to the official method as laid down in the manual of official test methods published by the Friedrich-Loeffler-Institut, the German federal research institute of Animal Health [18]. The question whether real-time PCR positive samples contained viable or non-viable MAP could not be answered in this setting. However, since the real-time PCR results as well as the culture results were used to determine the MAP status of the particular herd, and results were not used to determine the role of an infected environment on MAP transmission, the applied analysis is valid. 

Since test results were interpreted in parallel, test sensitivity was increased; therefore, we assume a high validity of the sensitivity for the whole sampling and testing approach used for the calculation of true prevalence. It was calculated from the data of a previous study using the same sampling approach and the same culture and PCR method [23]. The reference method in that study was a whole-herd testing of all cows by individual fecal culture. Analytical specificity as given by the manufacturer of the commercial PCR test was 1.0, which is not necessarily the diagnostic specificity. To account for a reduced diagnostic specificity due to pre- or postanalytical sample handling or laboratory contamination, we performed a sensitivity analysis using the values of 0.99 and 0.98, which is a common routine when using diagnostic tests, resulting in TP estimates, which were in the same range as calculated with a perfect specificity. Therefore, we conclude a high validity of our TP estimation. The nearly 80% difference between AP and TP reflects the 81% herd-sensitivity of the test approach when perfect herd-specificity is presumed.

The stratified random farm selection method used in this study ensured an acceptable level of external validity, i.e., representativeness for each region involved. The prevalence estimates presented here are valid for the respective target population, which are the dairy herds in the respective region. Although some of the respective regions with the relevant dairy industry were missing (e.g., the federal states North Rhine-Westphalia in the northern region, Baden-Württemberg in the southern region and Saxony in the eastern region), the provided prevalence estimations for the respective region are valid. The agricultural structure is comparable in the missing federal states of each region; therefore, results can be generalized for each particular region. Due to the limited number of herds, we have to accept the remarkable confidence intervals of the AP and TP estimators. Nonetheless, there is no overlap of the confidence intervals; therefore, they allow a clear distinction of different levels of between-herd prevalence. This supports our main conclusion that regions differ in their between-herd prevalence regarding MAP. Furthermore, this finding discourages the calculation of an average ‘national’ between-herd prevalence across the regions. This would even out MAP prevalence and thus not reflect the real situation regarding paratuberculosis infection in German dairy herds. 

A minor limitation of our study is the absence of two other relevant agricultural regions in Germany according to Merle et al. [13]. Since both regions comprise only small livestock populations with little relevance for the dairy sector in Germany these regions were not included. For the central region, a study was performed in Hesse in 2017 [10]. Boot swabs and PCR testing were used, but farms were not selected by stratified random sampling. Results showed an AP of 8.9% [10], which is between the determined MAP prevalence of RS and RN. 

Another limitation of our study arises from the disproportionality of sample size and number of herds in the study population. Although the southern region is characterized by a large number of small-sized herds, the eastern region includes a small number of large herds, with the northern region in between. Applying approximately the same sample size to these regions might result in an unequal variance for quantitative estimates, which could affect statistical comparison. However, the goal estimating the between-herd prevalence of different German regions was unaffected and could be reached by using the same methodology. Although the sample size was rather small, data are representative for the situation in each region since they were selected according to a random stratified sampling protocol per region. These estimates provide a robust basis for further epidemiological assessment regarding paratuberculosis. At the same time, they represent a starting point for future research or even national active monitoring on MAP prevalence in dairy cattle in Germany. 

## 5. Conclusions

From our results, we conclude that different approaches are needed to deal with the disease in regions with different between-herd prevalence ranging from on-farm prevalence reduction to mitigating the risk of spreading between herds. Measures for prevalence reduction and repeated herd status monitoring for success control are important for regions of high prevalence (east and north). Regions of low prevalence (south) should use those data to monitor MAP prevalence. Strategies reducing between-farm transmission of MAPs are important for all regions. Furthermore, a nationwide active monitoring would help to identify suitable control measures for all dairy production regions in Germany.

## Figures and Tables

**Table 1:** Herd characteristics of 457 German dairy herds from different regions.

	North (RN)	East (RE)	South (RS)
Herd numbers Herd size Median (Min, Max)	183 81 (12, 486)	170 199 (1, 2821)	104 38 (5, 206)
Breed (>80% HF *)	85.25	74.71	0.96
Housing (% free stall)	84.15	75.88	65.38

* HF = Holstein Frisian.

**Table 2:** Detection of viable *Mycobacterium avium* ssp. *paratuberculosis* (MAP) or MAP DNA in different kinds of environmental samples per region.

	Type of Sample	Total	PCR Pos	Culture Pos	Both
RN	Boot-swab	178	4	3	15 *
	Slurry	32	3	0	4
	Environmental fecal sample	157	2	2	9
RE	Boot-swab	169	17	5	41
	Slurry	60	8	4	16
	Environmental fecal sample	112	6	7	14
RS ^§^	Boot-swab	103	1	0	0
	Slurry	102	2	1	0

RN = Region North, RE = Region East, RS = Region South; ^§^ in RS only boot swab or slurry samples were collected. * one sample was culture-positive, but the internal amplification control of the real-time PCR failed to give a required signal (inconclusive PCR result).

**Table 3:** Number of positive farms and the apparent prevalence (AP) per region.

	Region North	Region East	Region South
*N*_(Farms)_	183	170	104
MAP-positive	22	69	3
MAP-negative	161	101	101
AP (%) [95% CI] ^$^	12.0 [8.1, 17.5]	40.6 [33.5, 48.1]	2.9 [0.1, 8.1]
TP (%) [95% CI] ^$^	14.8 [10.0, 21.6]	50.1 [41.4, 59.4]	3.6 [1.2, 10.0]
Sensitivity analysis Sp = 0.99 TP (%) [95% CI] ^$^	13.8 [8.9, 20.6]	49.5 [40.6, 58.9]	2.4 [0, 8.9]
Sensitivity analysis Sp = 0.98 TP (%) [95% CI] ^$^	12.7 [7.7, 19.7]	48.9 [39.9, 58.4]	1.2 [0,7.8]

N = Number, AP = Apparent Prevalence, CI = Confidence Interval, TP = True prevalence; ^$^ Confidence intervals are given as Wilson’s intervals for AP and Blaker’s intervals for TP [25].

## Data Availability

The data presented in this study are available on request from the corresponding author. The data are not publicly available due to written agreement between farmers and the research consortium regarding privacy of the farm’s MAP status.
